# Thymidine-dependent *Staphylococcus aureus* and lung function in patients with cystic fibrosis: a 10-year retrospective case-control study

**DOI:** 10.36416/1806-3756/e20240026

**Published:** 2024-08-07

**Authors:** Ana Paula de Oliveira Tomaz, Dilair Camargo de Souza, Laura Lucia Cogo, Jussara Kasuko Palmeiro, Keite da Silva Nogueira, Ricardo Rasmussen Petterle, Carlos Antonio Riedi, Nelson Augusto Rosario, Libera Maria Dalla-Costa

**Affiliations:** 1. Laboratório de Bacteriologia, Complexo Hospital de Clínicas, Universidade Federal do Paraná, Curitiba (PR) Brasil.; 2. Faculdades e Instituto de Pesquisa Pelé Pequeno Príncipe, Curitiba (PR) Brasil.; 3. Departamento de Análises Clínicas, Centro de Ciências da Saúde, Universidade Federal de Santa Catarina, Florianópolis (SC) Brasil.; 4. Departamento de Patologia Básica, Setor de Ciências da Saúde, Universidade Federal do Paraná, Curitiba (PR) Brasil.; 5. Departamento de Medicina Integrativa, Complexo Hospital de Clínicas, Universidade Federal do Paraná, Curitiba (PR) Brasil.; 6. Departamento de Pediatria, Complexo Hospital de Clínicas, Universidade Federal do Paraná, Curitiba (PR) Brasil.

**Keywords:** Staphylococcus aureus/drug effects, Thymidine/metabolism, Trimethoprim, sulfamethoxazole drug combination/adverse effects, Lung/physiopathology, Cystic fibrosis/complications

## Abstract

**Objective:**

Thymidine-dependent small-colony variants (TD-SCVs) of Staphylococcus aureus are being isolated with increasing frequency from patients with cystic fibrosis (CF). The aim of this study was to evaluate the relationship between TD-SCV isolation and pulmonary function in patients with CF, as well as to determine whether the emergence of TD-SCVs was associated with trimethoprim-sulfamethoxazole (TMP-SMX) use and with coinfection with other microorganisms.

**Methods:**

This was a retrospective case-control study including patients with CF who visited the Clinical Hospital Complex of the Federal University of Paraná, in Curitiba, Brazil, between 2013 and 2022. Demographic, clinical, and spirometric data, as well as information on TD-SCVs and other isolated microorganisms, were collected from the medical records of patients with CF and TD-SCVs (TD-SCV group; n = 32) and compared with those of a matched group of patients with CF without TD-SCVs (control group; n = 64).

**Results:**

Isolation of TD-SCVs was positively associated with TMP-SMX use (p = 0.009), hospitalization (p < 0.001), and impaired pulmonary function (p = 0.04).

**Conclusions:**

The use of TMP-SMX seems to contribute to the emergence of TD-SCVs, the isolation of which was directly associated with worse pulmonary function in our sample.

## INTRODUCTION

Cystic fibrosis (CF) is a multisystemic disease affecting the digestive, reproductive, and respiratory systems.^1^ In the respiratory tract, CF is associated with chronic obstructive bronchial disease caused by recurrent infections.^2^ This disease is caused by mutations in the CF transmembrane regulator (CFTR) gene, which encodes a CFTR protein that transports chloride ions outside cells.^3^ Because of the hypertonicity of chloride ions in the lungs, thick mucus favors colonization and infection by various microorganisms. Important pathogens in patients with CF include *Staphylococcus aureus*, *Pseudomonas aeruginosa*, *Burkholderia cepacia* complex, *Stenotrophomonas maltophilia*, and *Achromobacter xylosoxidans.* ^4^ ^,^ ^5^

It is not uncommon for *S. aureus* to be implicated in initial pulmonary infections in patients with CF.^6^ In such patients, small-colony variants (SCVs) of *S. aureus* are being isolated with increasing frequency.^7^ ^,^ ^8^ Colonies of SCVs arise because of multiple conditions that lead to the induction and selection of this phenotype, including auxotrophism caused by genetic mutations that determine the dependence on hemin and menadione due to aminoglycoside use and on thymidine due to trimethoprim-sulfamethoxazole (TMP-SMX) use.^9^ Due to this metabolic deficiency, it is difficult to identify *S. aureus* SCVs by routine laboratory microbiology. The colonies grown are 10-fold smaller than normal, non-pigmented, and non-hemolytic on blood agar. SCVs may also produce false-negative results on catalase and coagulase tests and fail to grow on Mueller-Hinton agar.^8^ ^,^ ^9^

The use of TMP-SMX contributes to the introduction of mutations in the *thyA* gene of *S. aureus*; this gene encodes thymidylate synthase, an essential enzyme for bacterial DNA synthesis. Those mutations characterize the thymidine-dependent SCV (TD-SCV) phenotype.^10^ ^,^ ^11^ In some isolates, the SCV phenotype is reversed to a normal phenotype, which may be related to persistent infections ^(^ ^12^ and treatment difficulties.^6^ Some studies have demonstrated that coinfection of TD-SCVs with other microorganisms, such as *P. aeruginosa*, *S. maltophilia*, and *A. xylosoxidans*, can worsen lung function.^4^ ^,^ ^13^

To evaluate pulmonary function, spirometry is performed in individuals with CF who are over five years of age.^14^ A decline in FEV_1_, as measured on spirometry, indicates a higher risk of hospitalization and death.^15^

Given that CF is associated with high morbidity and mortality in young patients and is often linked with recurrent infections, improving the understanding of *S. aureus* TD-SCVs and their clinical role may contribute to improving the prognosis of patients with the disease. This study was conducted to investigate the relationship between TD-SCV isolation and pulmonary function in patients with CF. In addition, we evaluated whether TMP-SMX use is related to the emergence of TD-SCVs. 

## METHODS

### Study design

A total of 286 patients visited the CF clinic of the Clinical Hospital Complex (CHC) of the *Universidade Federal do Paraná* (UFPR) in the city of Curitiba, Brazil, between July 2013 and September 2022. Of those, 96 patients with CF were selected for this case-control study. The presence of TD-SCVs was determined by analyzing respiratory tract cultures (sputum or oropharyngeal swab samples). The TD-SCV group (n = 32) comprised patients with at least one positive culture for TD-SCVs, whereas the control group (n = 64) comprised patients with CF who had a negative culture for TD-SCVs (ratio 1:2). Control group subjects were selected, on the basis of sex and age, from among other patients with CF in whom *S. aureus* had been isolated and who were coinfected with another microorganism.

### SCV identification and auxotrophic characterization

Respiratory tract samples were cultured on mannitol salt agar and blood agar. Smaller colonies were identified as *S. aureus* by using standard biochemical tests, and identification was confirmed with matrix-assisted laser desorption ionization time-of-flight mass spectrometry (Bruker Daltonics, Billerica, MA, USA).^8^

The nutritional dependence of the SCVs was tested by inoculating a suspension adjusted to a 0.5 McFarland standard on Mueller-Hinton agar (Oxoid; Thermo Fisher Scientific, Waltham, MA, USA) supplemented with hemin, menadione, or thymidine (10, 25, and 100 µg/mL, respectively; Sigma-Aldrich, St. Louis, MO, USA).^16^ The plates were incubated under aerobic conditions at 35°C for 24-72 h. Isolates of *S. aureus* SCVs were characterized as nutritionally dependent when they grew on a specific substrate but did not grow in its absence.^8^

### Demographic, clinical, and microbiological data

Demographic and clinical data were obtained by reviewing patient medical records. Age, sex, the results of CF detection tests (for immunoreactive trypsinogen, sweat electrolytes, and CFTR mutations), bronchiectasis, pancreatic insufficiency, hospitalization, death, spirometric values, and TMP-SMX use were evaluated. To determine the frequency of coinfection or colonization between other microorganisms and TD-SCVs, we evaluated *S. aureus*, *P. aeruginosa*, *B. cepacia* complex, *A. xylosoxidans*, and *S. maltophilia*, which were isolated at the time of their emergence.

Pulmonary function was evaluated by determining the percent of predicted FEV_1_ (FEV_1_%). The FEV_1_ values were obtained from spirometric tests performed closest to time of isolation of TD-SCVs, with a maximum period of one year before TD-SCV isolation. The control group comprised patients infected with *S. aureus* with a normal phenotype.

Pulmonary function testing was performed according to the standards established by the American Thoracic Society. Pulmonary function was categorized on the basis of the FEV_1_%, as follows^17^: normal, ≥ 90%; mild impairment, 70-89%; moderate impairment, 40-69%; and severe impairment, < 40%.

### Statistical analysis

Statistical tests were employed to evaluate demographic, clinical, and spirometric data, as well as the isolated microorganisms. Descriptive data were analyzed as absolute and relative frequencies for qualitative variables and as medians (ranges) for quantitative variables. Data normality was investigated with the Shapiro-Wilk test, and associations between variables were detected with Pearson’s chi-square or Fisher’s exact tests, as appropriate. In addition, differences between quantitative variables were evaluated with Mann-Whitney U tests. The significance level was set at 5% (*p* < 0.05). Data were analyzed with R software (R Core Team, 2022), version 4.2.1.^18^

This study was approved by the Institutional Ethics Review Board of the CHC/UFPR (Reference no. 45063115.9.0000.0096). The requirement for informed consent was waived because of the retrospective nature of the study.

## RESULTS

**Figure 1:**
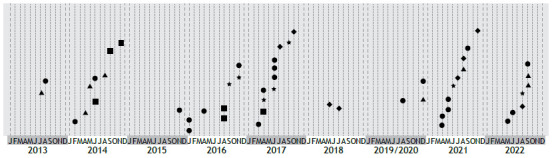
Timeline of the appearance of thymidine-dependent small-colony variants (TD-SCVs) in patients with cystic fibrosis during the study period. Number of times that TD-SCVs were isolated in each patient (P): ●only once (P2, P3, P5, P7-P10, P13-P18, P20, P22-P26, and P29-P32); ▲twice (P1, P4, P21, and P28); ■three times (P6 and P11); ♦four times (P19 and P27); and ★seven times (P12).

Among the 286 patients treated at the CF clinic of the CHC/UFPR, *S. aureus* was isolated from respiratory samples in 254 (88.8%) and TD-SCVs were isolated in 36 (14.2%). In 23 patients, TD-SCVs were isolated only once, whereas in nine patients, they were isolated multiple times over the 10-year follow-up period, as shown in Figure 1.

Demographic data and clinical characteristics of the patients, by group, are shown in Table 1. The table also shows the association between the emergence of TD-SCVs and TMP-SMX use; hospitalization rates; and coinfection with other microorganisms. The mutation most commonly identified among patients in the TD-SCV and control groups was the F508del mutation, followed by the G542X mutation. We found no association between the isolation of other microorganisms and coinfection with TD-SCVs. We detected a positive association between previous use of TMP-SMX and isolation of a TD-SCV. The proportion of patients that had been hospitalized was higher in the TD-SCV group (Table 1). Isolation of a TD-SCV was also associated with lower pulmonary function (FEV_1_%), as shown in Table 2.

**Table 1:** Demographic and clinical characteristics of patients with cystic fibrosis, with and without thymidine-dependent small-colony variants of *Staphylococcus aureus*.

Characteristic	TD-SCV group	Control group	p-value
	n/N (%)	n/N (%)	
Male	16/32 (50.0)	32/64 (50.0)	1.00
CFTR gene mutation^a^			
F508del	17/29 (58.6)	39/60 (65.0)	0.653
Homozygous F508del	3/17 (17.6)	17/39 (43.6)	0.205
G542X	7/29 (24.1)	24/60 (40.0)	0.100
Homozygous G542X	3/7 (42.9)	6/24 (25.0)	0.163
Other	7/29 (24.1)	6/59 (10.2)	0.109
Pancreatic insufficiency	28/32 (87.5)	57/64 (89.0)	1.00
Bronchiectasis	27/32 (84.4)	53/64 (82.8)	0.768
Hospitalization	14/32 (43.8)	6/64 (9.4)	< 0.001
Death	3/32 (9.4)	1/64 (1.6)	0.106
Use of TMP-SMX^a^	28/32 (87.5)	39/64 (60.9)	0.009
TD-SCVs and coinfection			
*P. aeruginosa*	13/32 (40.6)	22/64 (34.4)	0.708
*B. cepacia* complex	5/32 (15.6)	3/64 (4.7)	0.112
*A. xylosoxidans*	4/32 (12.5)	3/64 (4.7)	0.217
*S. maltophilia*	3/32 (9.4)	2/64 (3.1)	0.329

TD-SCV: thymidine-dependent small-colony variant; and TMP-SMX: trimethoprim-sulfamethoxazole. ^a^Data not available for all patients.

**Table 2:** Descriptive data analysis of demographic and clinical characteristics of patients with cystic fibrosis, with and without thymidine-dependent small-colony variants of *Staphylococcus aureus*.

Variable	Group	N	Range	Median	SD	p-value
Age (years)	TD-SCV	32	1-26	14.0	6.2	0.897
	Control	64	1-25	14.5	6.0	
Spirometry^a^						
FEV_1_%	TD-SCV	25	16-132	61.8	24.5	0.04
	Control	47	30-131	78.7	27.6	

TD-SCV: thymidine-dependent small-colony variant. ^a^Spirometric data not available for all patients.

**Figure 2:**
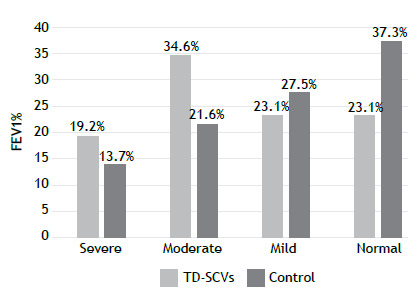
Classification of pulmonary function, by percent of predicted FEV_1_ (FEV_1_%), in patients with cystic fibrosis, with and without thymidine-dependent small-colony variants (TD- SCVs).

At the time of TD-SCV isolation, impaired lung function was more common among the patients in the TD-SCV group than among those in the control group. As can be seen in Figure 2, severe air flow obstruction was observed in 19.2% of the TD-SCV group patients (vs. 13.7% of the control group patients) and moderate air flow obstruction was observed in 34.6% of the TD-SCV group patients (vs. 21.6% of the control group patients). Pulmonary function impairment was found to correlate with the isolation of TD-SCVs (Table 2 and Figure 2).

## DISCUSSION

This case-control study involved patients at a referral center for CF who were colonized by or infected with TD-SCVs. The results show that the presence of these microorganisms was associated with worse lung function and a greater risk of hospitalization. These findings suggest that TD-SCVs, which are emerging pathogens associated with chronic infections, can influence the course of the disease over time.

During the study period, the prevalence of the TD-SCV phenotype in our sample was 14.2%, similar to that reported by Morelli et al. ^(^ ^19^ In our study, the median age at TD-SCV emergence was 14.0 years, comparable to the 14.4 years found by Yagci et al. ^(^ ^20^ but considerably lower than the 23.0 years found by Morelli et al. ^(^ ^19^ Those differences might be related to the age groups studied.

In the present study, the clinical data on pancreatic insufficiency were not positively associated with bronchiectasis. However, we observed an association between hospitalization rates and TD-SCV isolation. The higher morbidity observed in this group was likely related to greater impairment of lung function, as reported by Wolter et al. ^(^ ^7^ Although the number of deaths was higher in the TD-SCV group than in the control group, this difference was not significant. According to the literature, the association between TD-SCV isolation and the risk of death is related to this phenotype and to bacteremia. ^(^ ^5^

In agreement with previous studies, ^(^ ^10^ ^,^ ^21^ ^,^ ^22^ TMP-SMX use was significantly higher in our TD-SCV group, suggesting that the use of this medication is a risk factor for the emergence of the TD-SCV phenotype. This antimicrobial agent is commonly used to treat infections with *B. cepacia* complex, *S. maltophilia*, and methicillin-resistant *S. aureus*,^13^ all of which are commonly isolated from patients with CF.^17^ The protein thymidylate synthase is disrupted by *thyA* mutations caused by use of TMP-SMX, and that induces the production of high levels of cyclic di-AMP, which in turn induces activation of the interferon-stimulating protein in the host and increases the number of inflammatory cells in the airways, resulting in severe pulmonary dysfunction.^23^

Colonization by different microorganisms was used as a selection criterion for the control group; therefore, the time since TD-SCV isolation (coinfection) was not significantly different between the two groups. However, impaired pulmonary function (low FEV_1_%) was associated with the presence of TD-SCVs, which agrees with the results obtained by Wolter et al.^7^

Finally, it is unlikely that our findings were influenced by the CFTR genotype, because most of the patients in both groups had F508del and G542X mutations, the most common genotypes in our sample. However, our study has some limitations, such as the small number of samples and the limited amount of secondary clinical data. However, *S. aureus* infection with the TD-SCV phenotype is still underdiagnosed in patients with CF, and few studies have demonstrated the impact of infections with this phenotype.

In our study sample, TMP-SMX use was found to have affected the emergence of the TD-SCV phenotype. In addition, there was a direct relationship between worse pulmonary function and colonization/infection with TD-SCVs. Successful TD-SCV detection in microbiology laboratories is essential for adequate treatment, which affects morbidity and mortality in patients with CF.
